# Analysis of a two-year independent screening effort for retinopathy of prematurity in rural Egypt

**DOI:** 10.1186/s12886-021-02193-x

**Published:** 2021-12-27

**Authors:** Sara Tawfik, Ahmed Mansour, Norhan Lotfy Selim, Ahmed M. Habib, Yousef A. Fouad, Mohamed A. Tawfik, Mariam Al-Feky

**Affiliations:** 1 Al Ferdaws Eye Hospital, Sharkia, Egypt; 2 Al Mashreq Eye Center, 102 El-Sayed El-Merghany St., Cairo, 11774 Egypt; 3 Department of Ophthalmology, Ain Shams University Hospitals, Cairo, Egypt; 4 Memorial Institute for Ophthalmic Research, Giza, Egypt; 5 Watany Eye Hospital, Cairo, Egypt

**Keywords:** Retinopathy of Prematurity, ROP, Screening, Aggressive Posterior Retinopathy of Prematurity, Ranibizumab

## Abstract

**Background:**

The third epidemic of retinopathy of prematurity (ROP) has majorly involved middle income countries in which tailored screening and local guidelines require development. The data regarding ROP prevalence and cutoff numbers for screening in Egypt are lacking.

**Methods:**

Retrospective analysis of an independent screening effort spanning 2 years (February 2019 to February 2021) and involving 32 neonatal care units within Sharkia governorate, Egypt. Infants of gestational age (GA) ≤ 34 weeks and/or birth weight (BW) ≤ 2000 g were included, as well as those with unstable clinical course. Two eyecare centers located in Sharkia and Cairo governorates served as referral centers for any required interventions.

**Results:**

Of the 276 screened infants, 133 (48.2%) had some form of ROP that was bilateral in 127 (95.5%) of them. Aggressive posterior ROP (AP-ROP) was detected in both eyes of 24 infants (8.7%). The median (IQR) GA of infants with ROP was 32 (30–34) weeks, and the median (IQR) BW was 1600 (1350–2000) g. Sixty-three infants (47.4%) required treatment. Of the total 84 eyes that primarily were treated, 73 (86.9%) received intravitreal ranibizumab, 8 (9.5%) underwent laser ablation therapy, and 3 eyes (3.6%) underwent surgery. Recurrence rate was 16.7% (14 eyes). Final outcome was favorable in 83 eyes (98.8%). Applying the American Academy criteria would have led to the missing of 36.8% of infants with ROP and 28.6% of those requiring treatment in our sample.

**Conclusion:**

The incidence of both ROP and AP-ROP in the Egyptian rural setting appears to be in the high end of global reported rates. Prevention measures should urgently be planned and implemented.

## Background

Retinopathy of prematurity (ROP) is a leading cause of childhood blindness, especially in countries with rapidly-developing economies where preterm births are increasingly associated with survival [1]. The condition occurs in premature infants of low birth weight (BW) due to an arrest in normal neurovascular development of the retina, leading to aberrant vascular proliferation that has the potential to cause blindness [2]. With the availability of effective therapeutic options, early detection and proper management of ROP have been shown to prevent progression and to preserve vision [3].

The classification of the condition is based on the international classification of ROP (ICROP) [4] which localizes the disease to 1 of 3 retinal anatomical zones, and defines its extent according to 5 progressive stages. Plus disease, referring to venous dilation and arteriolar tortuosity in 2 or more quadrants of the posterior retina, is an additional sign that may accompany any stage of ROP and is important in defining treatment-requiring disease [5], with the term “pre-plus” often reserved for vascular abnormalities that are insufficient to meet the diagnostic criteria for plus disease [4]. Aggressive posterior ROP (AP-ROP) is often regarded as a standalone entity with a constellation of signs that reflect severe vascular abnormalities located in the posterior pole. Being rapidly progressive, the latter entity is often associated with extreme prematurity and suboptimal standard of care in neonatal intensive care units (NICUs), and accordingly varies in incidence between different settings [6].

Screening guidelines for ROP should be tailored for different countries as the cutoffs for gestational age (GA) and BW have been shown to vary [7, 8]. This is often attributable to difference in primary prevention, where NICUs in less equipped settings - especially rurally - lack sophisticated oxygen titration and measurement capabilities [8]. For example, the latest policy statement by the American Academy of Pediatrics and American Academy of Ophthalmologists (AAP/AAO) recommends screening infants of GA ≤ 30 weeks and/or BW ≤ 1500 g [9], while in India the screening cutoffs are set to a GA ≤ 34 weeks and/or BW ≤ 2000 g [10], highlighting the importance of proper documentation in each setting to develop tailored protocols that limit outlier cases [11]. This sequence has, however, been lacking in most developing nations; for instance, at the time of writing, only 3 countries in Africa (South Africa, Nigeria, and Kenya) have developed national screening guidelines and programs for ROP [12, 13].

The World Health Organization’s latest publication on global preterm birth [14] classifies Egypt as a group C country with insufficient birth registration data to reach exact figures on preterm birth, but puts the estimate at the regional average of 13.4% out of the country’s estimated 2.48 million live births in 2014. Scarce reports describing scattered efforts of ROP screening exist in the literature [15–17] but the country still lacks local guidelines and a national screening program [12].

In this work, we analyze a two-year independent screening effort for ROP in rural Egypt in an attempt to understand the affected population’s characteristics and needs within a regional and global context.

## Methods

This retrospective analysis included data from ROP screening conducted independently by our group in a rural setting in Egypt over the period of 2 years, from February 2019 to February 2021. The effort was directed towards infants that met the inclusion criteria for screening in 32 NICUs from 9 different districts in the outskirts of Sharkia governorate, in addition to limited direct referrals by neonatologists in private practice in the same region. The size of the NICUs ranged from those accommodating 3 beds to those accommodating 30 beds and the level of facilities ranged from those equivalent to an AAP level I NICU up to some elements of a level III NICU [18]. Two equipped eye centers in Sharkia (Al Ferdaws Eye Hospital) and Cairo (Al Mashreq Eye Center) served as referral locations for any additional interventions that were required, with facilities that included indirect ophthalmoscopes, 30-diopter lenses, pediatric eye speculums and indenters, a contact fundus camera (3nethra neo, Forus Health, India), equipped operating rooms with anesthesia staff experienced in neonatal sedation and general anesthesia, indirect laser photocoagulation via 810 nm diode laser (OcuLight SLx, IRIDEX, United States), cryotherapy, anti-vascular endothelial growth factor (anti-VEGF) injections, and experienced pediatric retina surgical staff.

Since there are no local guidelines for ROP screening in Egyptian newborns, the most inclusive ones were adopted by our team to limit the missing of cases. Infants of GA ≤ 34 weeks and/or BW ≤ 2000 g were included in the screening. Infants who were above these cutoffs but suffered unstable clinical course during NICU care, received inotropic support or oxygen supplementation for more than 2 days (especially if saturation was not regularly monitored), or those whose history during NICU admission was unknown or unclear were also included. Both the NICU staff and the parents were informed that the infant required ophthalmological evaluation at 28 days post-gestation. Infants who were still under NICU care on the screening day were examined in the NICU using portable indirect ophthalmoscopy, while those discharged had their parents instructed to bring them to one of the designated referral centers - or any equivalent eyecare facility - on the specified follow up date.

Screening technique, follow up schedules, and decision to treat were all based on the latest AAP/AAO recommendations [9], with staging of the condition based on the ICROP [4]. Topical anesthesia was used to examine pre-dilated eyes in infants placed in a swaddling position. Baseline characteristics and findings together with the clinical decision and follow up schedule were all documented in an electronic medical records system. Eyes with equivocal findings were photographed using fundus imaging and shared with the rest of the screening team for proper staging and clinical decision. Treatment was offered for eyes with type I disease (which includes any stage with plus disease in zone 1 or 2, and stage 3 in zone 1 without plus disease) as well as eyes with AP-ROP. Off-label use of intravitreal ranibizumab (IVR, 0.2 mg) injection was offered as the first line of therapy for type 1 disease in the NICU setting or at either referral center, since ranibizumab - but not bevacizumab - is approved for ocular usage by national health regulatory agencies. Ablation therapy via head-mounted laser indirect ophthalmoscope was offered primarily for cases with significant traction (advanced stage 3) and as a secondary intervention when persistence of avascularity was noted despite 3 doses of IVR. Surgical intervention was reserved for stages 4 and 5 of the disease. Both laser therapy and experienced surgical staff were only available in Cairo, to which the parents of requiring infants were referred.

Data were collected, revised, and tabulated in an Excel sheet. The Statistical Package for Social Science (IBM SPSS Version 25) was used for statistical analysis. The Shapiro-Wilk test was applied to measure departure from normality, with the data described in terms of mean (± SD) in case of normal distribution, and median with interquartile range (IQR) in case of non-normally distributed data.

## Results

The screening sample included 276 infants, of which 157 (56.9%) were males. The median (IQR) duration of NICU stay was 20 (12–30) days. The median (IQR) GA of the screened infants was 33 (31–35) weeks, while their median BW was 1700 (1400–2200) g. The median (IQR) duration to the first screening examination was 36 (29–60) days.

**Fig. 1:**
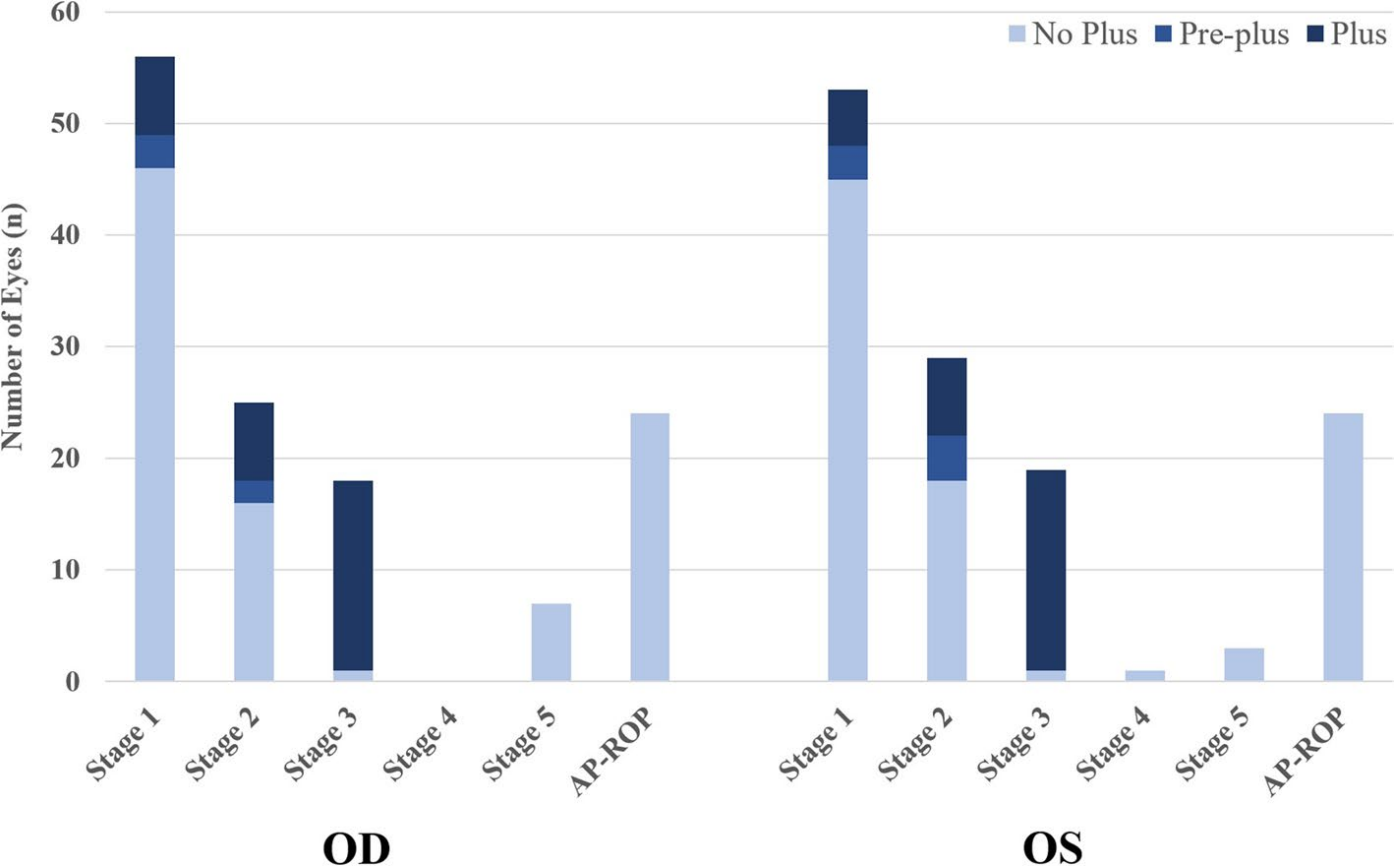
Distribution of ROP staging in both eyes of the screened infants (total screened infants *n* = 276, ROP diagnosed *n* = 133)

Of the screened infants (*n* = 276), 142 (51.5%) had fully vascularized retinas on initial examination and were discharged from screening. One infant (0.4%) had probable familial exudative vitreoretinopathy (based on an elder sibling’s history of the disease that had been confirmed by genetic testing abroad) with bilateral total retinal detachments, the infant was referred for genetic testing abroad due to its local unavailability, and for surgical intervention. One hundred and thirty-three infants (48.2%) had some form of ROP (Fig. 1) that was bilateral in 127 (95.5%) of them. Plus disease was detected in at least 1 eye of 33 infants (12%), while AP-ROP was detected in both eyes of 24 infants (8.7%). In infants with ROP, the male/female distribution was 73/60 (1.2:1), the median (IQR) GA was 32 (30–34) weeks, and the median (IQR) BW was 1600 (1350–2000) g.

Of the 133 infants with ROP, 63 (47.4%) required treatment (Table 1) and 70 (52.6%) required follow up. One infant (0.8%) died due to complications related to prematurity, while 17 (12.8%) were lost to follow up or refused treatment. Of the total 84 eyes that were treated, 73 eyes (86.9%) received primary IVR, with 9 eyes (10.7%) requiring subsequent injections for recurrence (range: 1–3 injections), 8 eyes (9.5%) underwent primary laser ablation therapy while 6 other eyes (6.8%) received secondary laser therapy due to incomplete vascularization after IVR, and 3 eyes (3.6%) underwent primary surgical intervention. The total recurrence rate was 16.7% (14 eyes). Final outcome was favorable in 83 eyes (98.81%). Only one infant (1.6%) had asymmetric progression in the left eye despite IVR.

**Table 1:** Screened infants requiring treatment, primary and secondary treatment modalities, and outcomes

Total Requiring Treatment	63	
Refused or Lost Follow Up	15 (23.8%)	
Death^a^	1 (1.6%)	
Received Treatment	47 (74.6%)	
	**OD**	**OS**
Primary Treatment	44	40
*Intravitreal Ranibizumab*	38 (83.4%)	35 (87.5%)
*Laser Ablation*	4 (9.1%)	4 (10%)
*Surgery*	2 (4.5%)	1 (2.5%)
Recurrence	7 (15.9%)	7 (17.5%)
Secondary Treatment		
*Intravitreal Ranibizumab*	5 (11.4%)	4 (10%)
*Laser Ablation*	3 (6.8%)	3 (7.5%)
*Surgery*	0 (0)	1 (2.5%)
Final Outcome		
*Full Vascularization*	40 (90.9%)	35 (87.5%)
*Arrested Vascularity*	4 (9.1%)	4 (10%)
*Progression of ROP*	0 (0%)	1 (2.5%)

^a^By prematurity complications

If we apply the AAP/AAO GA and BW criteria for screening on our sample, 126 of the 276 screened infants (45.7%), 49 of the 133 infants with ROP (36.8%), and 18 of the 63 infants requiring treatment (28.6%) would fall outside the criteria.

## Discussion

The third epidemic of ROP mostly involves middle-income countries, where wider NICU availability is increasingly supporting the survival of infants, but suboptimal care and improper oxygen administration and monitoring are resulting in higher rates of ROP among older and heavier infants [19, 20]. This is especially true in rural settings where higher rates of more severe forms of the disease are reported [21, 22]. In this work, we have attempted to explore the incidence and behavior of ROP in a cohort of Egyptian premature infants within a rural setting, highlighting the sample’s characteristics and challenges in evaluation and management.

The incidence of ROP in our sample (48.2%) is higher than rates reported in studies from other middle income countries like India (Eastern India 33.2% [23], Northern India 26.6% [24], and rural outreach centers 22.4% [25]), Turkey (30% [26] and 27% [27]), South Africa (29.6% [28]), Iran (23.5% [29]), Palestine (23.5% [30]), and Botswana (11% [31]). The incidence of AP-ROP (8.7%) is also beyond the high end of the spectrum of rates reported in the literature that range from 0.1 to 5% [6]. Both findings reflect on the iceberg burden of ROP in Egypt, the suboptimal quality of maternal and neonatal healthcare in the country’s rural setting, and the dire need for a national prevention program.

The delay in initial screening in our sample (IQR: 29–60 days) could have contributed to the higher rates of both full retinal vascularization and severe disease on initial examination. In our setting, the burden of meeting the screening schedule rested solely on the parents. A study in an Indian population [32] reported that the main barriers to early screening consisted of a triad of unavailability of sufficient trained ophthalmologists, lack of awareness among parents and healthcare personnel, and distance from point of care. Another study [33] even reported that, in a rural setting, initiating screening on first contact with the infant in the NICU before the recommended time of conventional screening (21–28 days) ensured a better yield of screened infants, better pre-counselling of mothers, and higher rates of enrollment in and compliance to screening schedules. This highlights our setting’s need for more specialized, trained ophthalmologists and for comprehensive awareness campaigns to ensure wider, timely coverage of the screening process.

Four independent studies on ROP incidence in an Egyptian setting exist to date in the literature. Abdel Hakeem et al. [15] and Nassar [17] screened for ROP in a single NICU setting within a university hospital in upper Egypt and found the incidence of the condition to be 19.2 and 36.5%, respectively. Ezz El Din et al. [34] and Hadi et al. [16] examined the incidence in the more urban settings of a Cairo university hospital and 3 Alexandrian private hospitals and found it to be 18.9 and 34.4%, respectively. It is imperative to mention that all the aforementioned studies utilized the lower cutoff GA of 32 weeks and BW of 1500 g (except Hadi et al. [16] who used 1250 g as the cutoff) for screening, and none reported on AP-ROP incidence. Screening cutoffs should be tailored to each setting [8], and it has previously been established that ROP in developing countries could affect older and heavier infants, such that in these settings it would be rational to adopt more inclusive or earlier screening criteria [35, 36]. In our sample, applying the lower cutoffs of the AAP/AAO criteria would have led to the missing of 36.8% of infants with ROP and 28.6% of those requiring treatment.

IVR was widely offered for patients requiring treatment in our sample due to the convenience of availability and easier technicality of administration. This is in line with the reported overreliance of developing nations on anti-VEGF to treat ROP [8]. To date, the largest randomized trial by Stahl et al. [37] reported that IVR (0.2 mg) might be superior to laser therapy when it comes to unfavorable ocular outcomes, and that it processed a good 24-week safety profile. Furthermore, Barry et al. [38] have recently reported that IVR administered to infants using bedside sedation results in faster return to pre-procedure respiratory baseline than laser ablation therapy under GA.

Limitations to our work include the relatively small sample size, especially when considering the large, estimated burden of premature births in the country. Being specific to the rural settings, our results may also be non-generalizable to a national level, and future well-designed studies in different settings are needed to complement our work. Nevertheless, we have intended for the work to serve as a preliminary report, ushering organized efforts for screening and prevention of a condition of national and global public health relevance.

## Conclusion

In conclusion, Egypt appears to be very much involved in the third epidemic of ROP. Prevention measures should urgently be planned and implemented.

## Data Availability

Data are available upon request to corresponding author.

## Abbreviations

AAP/AAO: American Academy of Pediatrics and American Academy of Ophthalmologists
AP-ROP: Aggressive posterior Retinopathy of Prematurity
Anti-VEGF: Anti-Vascular Endothelial Growth Factor
BW: Birth Weight
GA: Gestational Age
ICROP: International Classification of Retinopathy of Prematurity
IQR: Interquartile Range
IVR: Intravitreal Ranibizumab
NICU: Neonatal Intensive Care Unit
ROP: Retinopathy of Prematurity
SD: Standard Deviation
